# Factors contributing to food choice in the UK secondary school food setting: a systems map perspective

**DOI:** 10.1017/S136898002510147X

**Published:** 2025-12-03

**Authors:** Niamh O’Kane, Ruth Hunter, Desiree Schliemann, Leandro Garcia, Jayne Woodside

**Affiliations:** Centre for Public Health, School of Medicine, Dentistry and Biomedical Sciences, Queen’s University Belfast, Belfast, UK

**Keywords:** Food choice, School food, Children, Systems, Systems map, Causal loop diagram

## Abstract

**Objective:**

To co-develop a systems map of the UK secondary school food system and to understand what factors contribute to food choice within it.

**Design:**

Participatory methods were used with a range of UK school stakeholders to co-produce a systems map of factors contributing to food choice in the secondary school food system. An online survey with stakeholders (*n* 26) was used to gather an initial list of factors, and a group model-building workshop was conducted with stakeholders (*n* 13) to establish relationships between these factors. Two school workshops captured the views of students (*n* 17). The map then underwent final refinement by the research team, and all stakeholders were provided the opportunity to provide feedback on the final version.

**Setting:**

United Kingdom.

**Participants:**

UK school stakeholders.

**Results:**

The systems map contained twenty-four factors with forty-three direct causal relationships between them, each factor falling into one of six themes: catering and procurement; school leadership and governance; the priority of food within schools; social experience, behaviours and attitudes; the food space and experience in school and financial. The map demonstrates how each of the factors interacts with each other, including the direction of influence. It also reveals feedback loops that shape and sustain food choice patterns in secondary schools.

**Conclusions:**

The systems map provides a visualisation of the complex secondary school food system and can be used by stakeholders in the design and evaluation of whole-school, multi-component interventions and programmes targeting food choice in secondary schools.

Poor diet quality in children and adolescents is of utmost concern due to the many impacts it can have on the lives of children and adolescents. Poor diet quality can contribute to childhood obesity, associated with increased risk of non-communicable diseases later in life,^(1,2)^ and in children and adolescents is also associated with micronutrient deficiency, higher dental caries and poorer physical growth and development.^(3,4)^ Furthermore, an estimated 15 percent of UK households with children experience food insecurity, associated with poor quality diets and less physical activity.^(5–7)^ Thus, these dietary, and resulting health, inequalities disproportionately affect households of lower incomes and those in deprived areas.^(8)^

To address and improve these inequalities, it is important to consider what settings may be appropriate for intervention. Schools are settings with the capacity to significantly impact dietary quality and health equity, as ‘structured settings’ like schools influence routines, behaviours and weight-related health outcomes in children and adolescents.^(9,10)^ Emerging COVID-19 research suggests that school closures highlighted the crucial role of structured settings in shaping children’s health behaviours and weight-related outcomes.^(10)^ Furthermore, the WHO Health Promoting Schools framework promotes school health programmes as a strategic means to preventing health risks and advocates for whole-school approaches to promoting health.^(11,12)^ Therefore, schools are a significant potential setting for the improvement of diet quality and health inequalities; however, schools are large, complex organisations and thus we must consider the importance of whole-school approaches.

Whole-school approaches are those that reach beyond education within the classroom to encompass all aspects of the life of a school, including the culture of a school, to offer opportunities for improved healthy development.^(13–16)^ A whole-school approach to food would therefore require the involvement of all actors within the school community, including school staff, administration, students, teachers, parents and beyond.^(17,18)^ To co-design, develop and implement whole-school approaches to food, we first must understand the full system with all its stakeholders and interactions. Systems science can be used to better understand the complexity of the school food system, as it does not just focus on the components of a system, but helps us to focus on how components within a system are dynamically related.^(19,20)^ Systems thinking is relatively new within obesity prevention, but has been increasingly employed in recent years.^(11,21–23)^ A recent study produced a systems map of the primary school food system, which demonstrated benefits in illustrating the complex system within schools.^(11)^ Whilst there are some learnings from this work that can be applied, due to the difference in age and development stage of pupils, and differing school structures, there are benefits in additionally exploring the secondary school food system. Additionally, as secondary schools are often larger and more complex organisations than primary schools, it could be more challenging to implement whole-school approaches within these settings. Employing a systems lens to the secondary school food system can therefore help shed light on the many influential factors, and the interplay between them, contributing to food choice during the school day.

The UK Prevention Research Partnership-funded ‘Generating Excellent Nutrition in UK Schools’ (GENIUS) Network was established in 2020, bringing together key school stakeholders with the aim of working towards a more health promoting food and nutrition system in UK schools. One of the aims of the GENIUS Network was to use innovative research methodologies to understand how the secondary school food system operates as a complex adaptive system. The aim of this study was to employ systems thinking to co-develop a systems map of the secondary school food system, to answer the question: ‘what interrelated factors contribute to food choice in the UK secondary school food setting?’. The aim is that the resulting map, co-developed with a range of stakeholders, can be used to illustrate the secondary school food system and be used to explore impact of future systems-informed school-based interventions, programmes and policies.

## Methods

### Study design

To create a systems map inclusive of the experiences and perspectives of multiple stakeholders within the system (i.e. the UK secondary school food system), we employed a number of participatory methods: (i) an online survey targeted to stakeholders; (ii) an online group model building workshop with diverse stakeholder representation and (iii) workshops with pupils from two secondary schools. Details of these methods follow.

#### Online stakeholder survey

An online survey (see online supplementary material, Supplemental File 1) was hosted on Qualtrics (Qualtrics.com) and disseminated through the GENIUS School Food Network. The survey aimed to gather factors influencing food choice in secondary schools, to seed the systems map prior to the workshop. Approximately 250 GENIUS mailing list subscribers and 450 Twitter followers were invited to participate. Participants provided consent at the outset of the survey. Participants were asked to identify: their role within the school food system, where in the United Kingdom they were based, and, the factors (min 5–max 15) they believed contribute to food choice in a secondary school food setting. Participants then had the option to enter a prize draw for a £50 Amazon voucher.

The survey received twenty-five responses and 245 factors were identified (survey respondent roles and factors listed in full in online supplementary material, Supplemental File 2). These factors were collated and initially consolidated under broader thematic factor names (by researchers NOK and JW) to organise the data. These factors were inputted into the software being used in the group model building workshop (STICKE) as nodes.

#### Online group model building workshop

A group model building workshop, led by two experts in systems science (RH and LG), was conducted online. Survey participants who provided email addresses for future research were contacted, and wider invites were sent through the GENIUS School Food Network (mailing lists, Twitter). Thirteen stakeholders participated, including local government working with schools (*n* 5), parent (*n* 2), academic/researcher (*n* 2), public nutrition body (*n* 1), teacher (*n* 1), charity (*n* 1) and nutritionist working with schools (*n* 1). Pre-workshop, participants provided written consent and received preparatory materials including a list of the factors identified from the survey, an agenda and background information on the project.

The workshop overview is in online supplementary material, Supplemental File 3. It started with introductions, defining the problem and introducing systems thinking and causal loop diagrams. Participants reflected on survey factors and suggested missing ones. Facilitators guided them in connecting factors to generate the structure of the systems map. Participants were prompted to consider: i) which factors may be causally related within the system and ii) directionality of this relationship. For example, factor A and factor B may be related, but participants were asked whether it was a causal relationship important within the system, to suggest which one influences the other, and whether this influence is positive or negative (i.e. does an increase or improvement in factor A cause an increase or improvement in factor B, or does an increase or improvement in factor A cause a decrease or deterioration in factor B?).

The workshop resulted in an initial systems map (a causal loop diagram) of the UK secondary school food system. Factors influencing food choice being represented as nodes, and connections between nodes depicting causal relationships, showing influence direction and whether this was positive or negative.

#### School workshops

After creating the initial systems map, the team sought feedback from key stakeholders: pupils. Secondary schools in Northern Ireland were invited to participate via email, with NOK conducting preliminary calls to explain the study. Two schools participated (one Catholic secondary and one non-denominational grammar), each receiving a one-off £150 contribution, as a thank you. Pupils provided informed consent, and two in-person workshops were conducted and voice recorded, with a total of *n* 17 pupils aged 16+, lasting an average of 38 min. Pupils were provided with a printed version of the map, and they discussed the factors they believed to be most influential, their direction of influence and rated the map. Pupils were asked to rate the systems map, firstly, according to appropriateness of the information to ensure the information was presented in a way they believed appropriate and understandable and, second, according to its applicability to their school, their experience and experience of their friends and peers, to ensure that the map was reflective of the pupil experience. Their input was incorporated into the map and their feedback refined it further.

#### Final systems map refinement

The final refinement stage included identification of themes (and colour scheme), to improve readability and visual clarity in accordance with pupil feedback. Stakeholders from the online workshop were then contacted via email to review the updated map. Lastly, the authors with expertise in school food systems (JW, NOK) identified major feedback loops with the potential to influence whole-school impact (i.e. other loops were perceived as significantly less important to the internal dynamics). Feedback loops are cause-and-effect chains which connect two or more factors in a circuit (loop). Feedback loops can be positive (factors reinforcing each other over time) or negative (factors balancing each other over time).^(24,25)^

## Results

**Figure 1.**
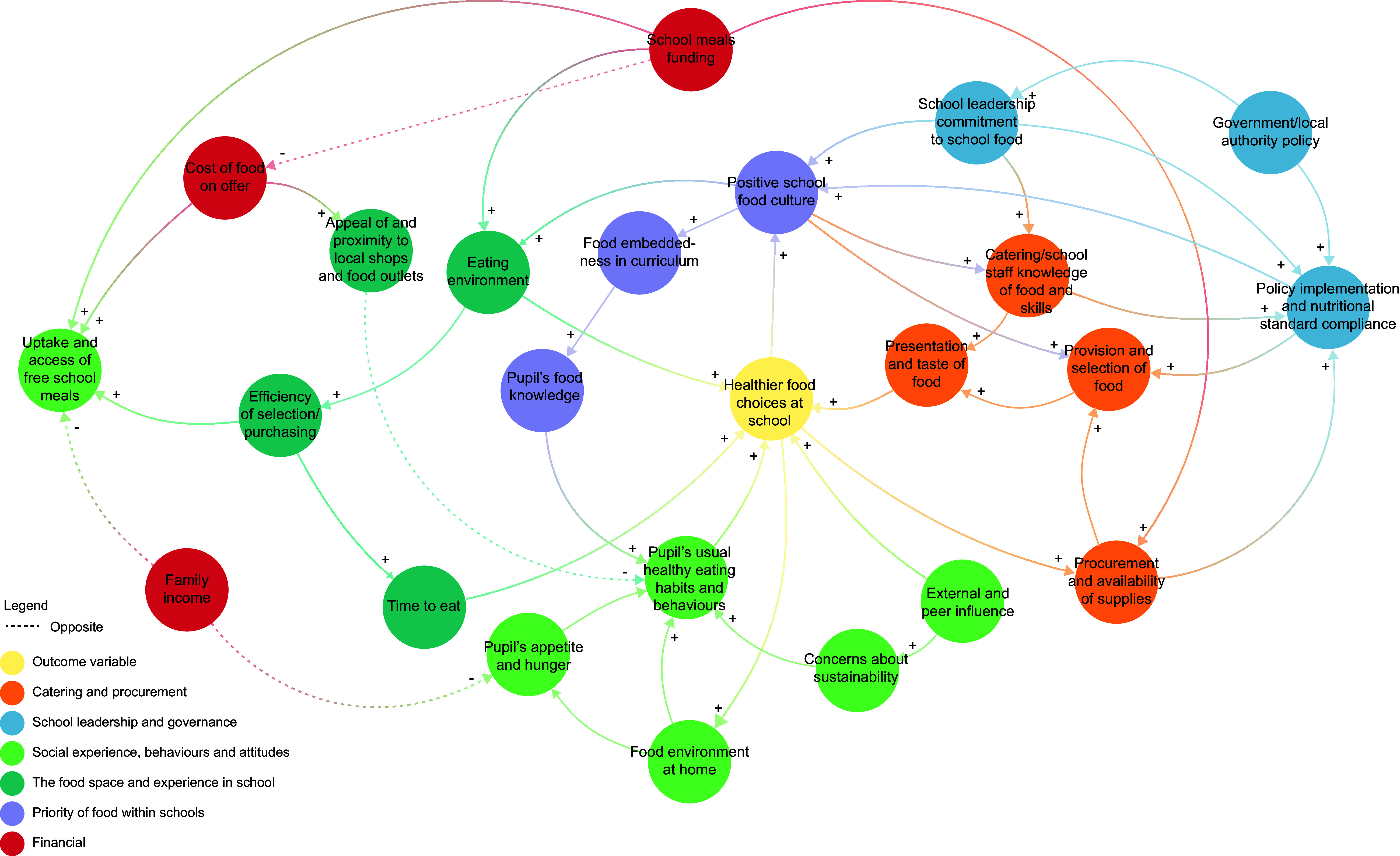
Systems map of factors contributing to food choice in the secondary school food system.

The systems map (Figure 1) draws on insights from a range of school stakeholders. The map contains twenty-four factors with forty-three direct causal relationships identified between them. A list of factors with descriptions can be found in online supplementary material, Supplemental File 4. ‘Healthier food choices at school’ sits centrally in the map and acts as an outcome variable within the school food system, that is, the desired outcome. The remaining factors fall within one of six themes: ‘catering and procurement’, ‘school leadership and governance’, ‘the priority of food within school’, ‘social experience, behaviours and attitudes’, ‘the food space and experience in school’ and, ‘financial’.

In this section, we present insights according to the themes identified within the map.

### Catering and procurement (orange nodes)

Four factors fell within the ‘catering and procurement’ theme: presentation and taste of food, catering/school staff knowledge of food and skills, provision and selection of food and procurement and availability of supplies.

Stakeholders identified that at the point of selection or consumption, presentation and taste of food will always play a crucial role in the food pupils will choose to eat. This encompassed several different considerations including portion size, food architecture, placement, labelling, nudging and marketing. It related to canteen food but also the additional opportunities for food presentation in schools (e.g. baskets of free fruit). Stakeholders identified that this is influenced by knowledge and skills of catering staff and other staff with roles in the food experience (e.g. teaching and support staff acting as break and lunch monitors). For example, caterers’ knowledge of appropriate portion sizes, or knowing how to place and nudge certain ‘healthier’ foods to encourage uptake. However, presentation and taste are also limited by the provision and selection of food, that is, the food that the school buys in. Caterers highlighted they often feel restricted with presentation in canteens because they are constrained by the ingredients and items they have to work with. This factor is therefore influenced by the procurement process and availability of supplies (as demonstrated in the map). Multiple stakeholders noted procurement framework limitations, including supplier restrictions and supply availability.

### School leadership and governance (blue nodes)

Three factors fell under the ‘school leadership and governance’ theme: policy implementation and nutritional standard compliance, school leadership commitment to school food and government/local authority policy.

Policy implementation and nutritional standards compliance (i.e. visibility and enforcement of a school food policy (e.g. packed lunch policy) and compliance with national nutritional standards) was a factor with multiple relationships to factors within the catering and procurement theme. The ability to successfully implement food policies relied upon the catering and school staff’s food knowledge and skills and by the restrictions placed on procurement, and the ability to successfully implement food policies impacted the provision and selection of food. It is also influenced by the government/local authority policy, that is, the policies and standards set upon the school by external bodies. The school leadership’s commitment to school food was a frequently reported factor driving food in schools, in both the online survey and the group model building workshop. That is, the commitment, passion or drive of the headteachers and other senior members of staff, in placing food as a high priority within the school. It was indicated by stakeholders that the headteacher sets the tone for the school and, if food was a priority, this could result in better training for staff, the creation and implementation of whole-school food policies and approaches and overall instil a more positive school food culture.

### Priority of food within schools (purple nodes)

Three factors fell within the ‘priority of food within schools’ theme: positive school food culture, food embeddedness in curriculum and pupil’s food knowledge.

A positive school food culture was identified as an important factor for multiple reasons. Having a positive ethos and culture around food in school acts as a bridge between school leadership and governance, catering and procurement and the food space and experience in schools. Stakeholders observed that healthier food choices by children create a positive school food culture, rather than the inverse. In such a culture, food is integrated into the curriculum, directly (e.g. home economics, food preparation and nutrition education) or indirectly (e.g. exploring food cultures in geography). Stakeholders, including pupils, agreed that integrating food into the curriculum can improve pupil’s general food knowledge. Pupils studying home economics felt more knowledgeable of food than their peers who didn’t study the subject.

### Social experience, behaviours and attitudes (light green nodes)

Six factors fell into the ‘social experience, behaviours and attitudes’ theme: pupil’s usual healthy eating habits and behaviours, external and peer influence, concerns about sustainability, food environment at home, pupil’s appetite and hunger and eligibility for and uptake of free school meals.

Within the map, pupil’s usual healthy eating habits and behaviours directly influence the food choices made at school, and these are also influenced by several factors. The home food environment affects how pupils approach food and their hunger and appetite levels at school. (i.e. if they have breakfast or have eaten well the night before). Stakeholders agreed that school food choices can influence the home food environment, as children may adopt good or bad habits from school. For example, informing their parent they tried something new and would like it at home, or purchasing soft drinks from vending machines in school, and wanting them at home. Stakeholders, including pupils, believed that pupils are forming their own opinions about food and developing their individuality around food, and of particular interest to the current generation is sustainability. For example, school and catering stakeholders reported a perceived increase in demand for vegan/vegetarian foods. It was believed that this is in large part due to external and peer influences, that is, peer behaviours, the pupil’s popularity, other children selling drinks and sweets purchased outside school, but also influences from the media, food advertising, and trends in food. Stakeholders believed these to influence food choices directly and indirectly. Finally, the ‘uptake of free school meals’ factor relates to both equity of access for pupils eligible for free school meals and potential stigma associated with free school meals and barriers this creates to uptake.

### The food space and experience in school (dark green nodes)

Four factors fell under the ‘food space and experience in school’ theme: eating environment, efficiency of selection/purchasing, time to eat and appeal of and proximity to local shops and food outlets.

The eating environment is a factor acting as a bridge between several themes and was a consistent factor raised across all research activities, particularly by the students. It refers to the space in which pupils eat, the access and speed of queues, noise, dining facilities, seating arrangements and the space allowed for consumption of food. Pupils and teachers recently recognised the true influence of the eating environment, highlighted by COVID-19 pandemic disruptions (e.g. staggered times, eating in classrooms and hand sanitiser queues). This linked to the issues with school food selection/purchasing processes, such as ordering methods, ease of payment and speed of access of the system. This had the potential to encourage or discourage pupils from availing of school food and potentially increase free school meal stigma. Inefficient systems also reduce eating time, influencing food choices. For example, pupils often preferred quick packed lunches to maximise lunchtime activities like football. The appeal of and proximity to local shops and outlets also impacts food choices, especially for older students who could leave school for lunch and was linked to cost of food on offer. For example, pupils reported preferring cheaper fast-food options, like a local McDonald’s, over more expensive school canteen meals.

### Financial (red nodes)

Three factors fell within the ‘financial’ theme: cost of food on offer, family income and school meals funding.

Factors within the ‘financial’ theme influence almost every other theme on the map. First, as discussed in the previous section, the cost of food on offer in most school canteens is deemed as too expensive for its quality or portion size by pupils and parents. This is influenced by the school’s funding for food, school meals and facilities, which also impacts on catering and procurement, the food space and experience in school and the resulting social experience. Also within the theme is family income, encompassing money available to pupils, school meal eligibility and food poverty. Family income is directly impacting upon the pupil’s appetite and hunger and the uptake and access of free school meals.

### Feedback loops

The major feedback loops within the systems map are identified and described in full in Table 1. This includes three reinforcing feedback loops (R1–R3). No balancing feedback loops were identified. The feedback loops incorporate many themes identified within the systems map, with an emphasis on the role of catering and procurement. Briefly, R1 illustrates the relationship between the food environment at home and healthier food choices at school. R2 illustrates the positive feedback loop of having a more school positive food culture, which can positively influence the eating environment and food choices at school, which will influence the demand and thus the supply of healthier food choices, which will help schools to comply with standards and policies, further perpetuating a positive school food culture. R3 illustrates the role of school and catering staff in influencing healthier food choices at school, positive school food culture and policy implementation and nutritional standard compliance.

**Table 1.** Main feedback loops identified within the systems map

Name	Feedback loop	Description
R1	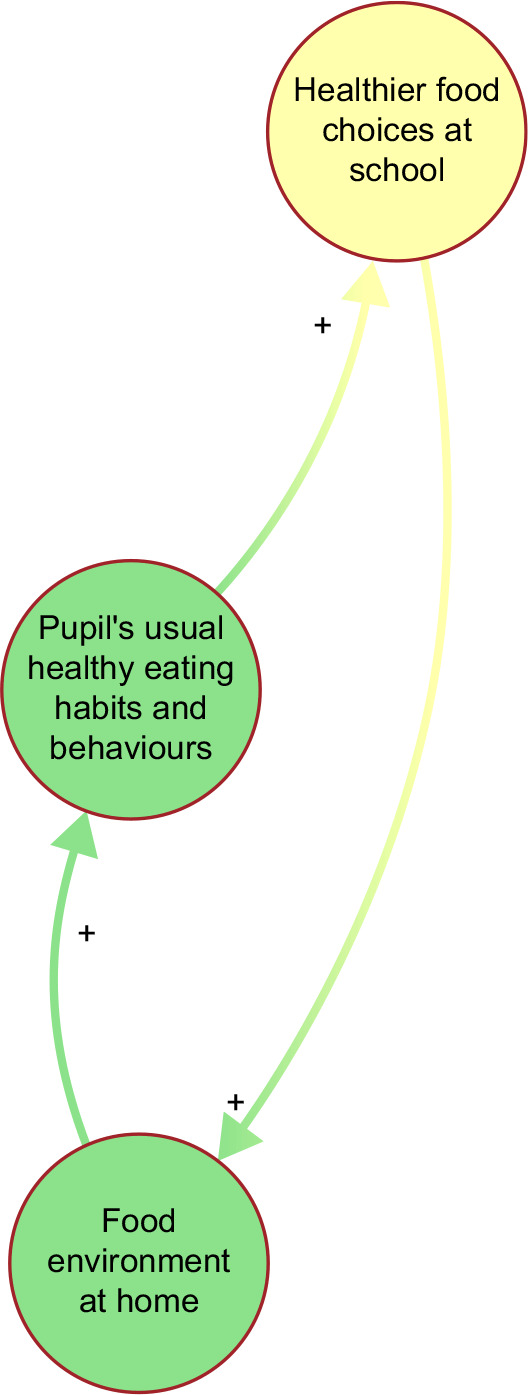	The food environment at home influences pupils’ usual eating habits and behaviours: the better the food environment at home, the better pupils’ healthy eating habits and behaviours might be, which can increase healthier food choices at school, which can then reinforce positive attitudes and behaviours at home.
R2	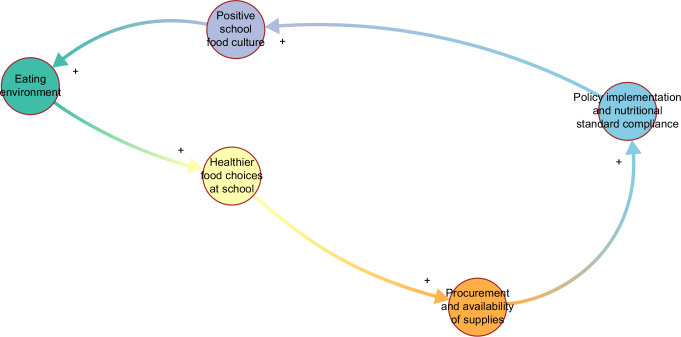	A school with a more positive food culture will be one with an improved eating environment, which can encourage healthier food choices at school. These healthier food choices providing increased demand of healthier foods at school can enable school leadership and catering teams to procure supplies for healthier food choices, which can increase chances of complying with nutritional standards and implementing nutritional policies.
R3	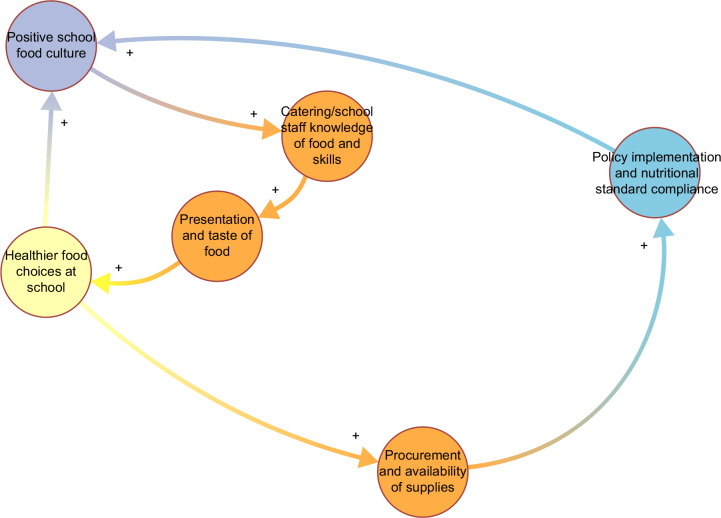	A school with well-trained catering and school staff in food and food skills will potentially produce food, which is presented and tastes better, which may encourage more healthier food choices at school. A higher demand for ‘healthier’ foods enables leadership and catering staffs to procure more of these foods, which can encourage better implementation of nutritional standards and healthy eating policies, which, when met, will contribute to a more positive school food culture. A school with a positive food culture will prioritise training of staff.

## Discussion

The study generated a systems map, co-developed using participatory methods with a range of stakeholder, illustrating the interactions between factors contributing to food choice in UK secondary schools. The map included twenty-four nodes with forty-three direct causal connections, representing factors and the relationships between them. The map covers various areas relating to the secondary school system, including catering and procurement, school leadership and governance, the priority of food within schools, social experience, behaviours and attitudes, the food space and experience in school and financial. The systems map reveals feedback loops that shape and sustain food choice patterns in secondary schools.

Many of the factors and relationships identified by stakeholders in the co-production of the systems map are supported by existing evidence in the literature, for example, the interplay between the home and school environment,^(26)^ the importance of good leadership practice and positive school culture^(16)^ and the impact of the school eating environment on food choice.^(27)^ School stakeholders understand the interconnected factors from their place within the system, but their experiences and perspectives vary. Creating a systems map such as this helps consolidate a shared understanding and can inform researchers about factors not yet identified or studied.

Viewing the secondary school food system through this systems lens allows us to appreciate the complexity of employing whole-school food approaches. The twenty-four factors sitting within six themes highlight the various components and dynamics at play within the secondary school food setting and the forty-three causal relationships that were identified demonstrate the potential widespread impacts of intervening in one small area in the system. Additionally, the secondary school food setting is host to a wide and diverse group of stakeholders, and a whole-school food approach requires buy-in from all stakeholders within the system, from school leadership and administration to staff and students, parents and beyond.^(18)^ Whole-school food approaches can have significant impact on dietary behaviours and food literacy, health and well-being of students and reduce food insecurity and inequalities.^(11,15,28–31)^ However, with whole-school food approaches, it is important that all stakeholders are all aligned in their objective and coordinated in their actions to ensure successful outcomes.^(17)^

The three key feedback loops in the systems map provide us with valuable insights into the dynamics of the school food system. They demonstrate that the school food system is not just composed of choices made within the school grounds but is inclusive of the home environment and the food choices and attitudes established outside of school. Feedback loop R1 reinforces the idea that the home environment influences the pupil’s usual healthy eating habits and behaviours, which influences how they behave around food at school.^(32)^ Healthier food choices at school can also be brought home and influence the broader food environment at home, demonstrating the potential widespread impact of whole-school food interventions which may benefit parents, siblings and the wider family unit. They also demonstrate the wide-reaching influence of a positive school food culture, with R2 illustrating the positive feedback between the demand and supply of healthier food choices at school and the resulting policy implementation and nutritional standard compliance. The benefits of a positive school food culture are well documented^(33,34)^ and the systems map further demonstrates that, whilst achieving a positive school food culture is a complex challenge, the benefits of it are reinforced within the wider school food system. Furthermore, the crucial role of the school and catering staff in influencing the system is demonstrated in feedback loop R3, further highlighting the need to prioritise the training, development and support of all school staff, including those in catering, support and teaching roles.^(35)^

Visualising the whole school food system provides insight into which stakeholders need to be involved, who needs to communicate with whom, possible leverage and resistance points and what anticipated and unanticipated consequences interventions can potentially cause. It demonstrates that food choice does not happen in a vacuum, but rather, it is impacted by and impacts the system in which it sits.^(21,36)^ The systems map can be used to identify priority actions which appear to have the potential for optimal reconfiguration of the system that shape food choice at school. It can also be used by actors external to the schools themselves, such as local authorities and policy makers, to understand how schools can be impacted by change in policy or local public health interventions.^(36)^ Therefore, this systems map can be used to aid the co-design and evaluation of whole-school, multi-component interventions and programmes targeting food choice in secondary schools.

The online methods employed in the survey and group model building workshop ensured the systems map was co-developed with stakeholders reaching across the four nations of the UK, allowing us to gather views from across the country. However, it is important to note that there are differences across secondary schools in the UK, in terms of food provision during the school day, school food policies and free school meal provision.^(37,38)^ For example, each UK nation has food-based standards implemented, but only Wales and Scotland also have nutrient-based standards.^(37)^ Particularly at the secondary level, schools, even within the same UK nation, will differ regarding lunch policies. Whilst all schools will have some provision of sit-down hot food, schools may differ in the level of less healthy choices on offer.^(39)^ Pupils may also be allowed to bring in packed lunches, which can have implications for diet quality, for example, higher intake of ultra-processed foods may be consumed via packed lunches.^(40)^ Furthermore, some secondary schools will allow all or a small number of year groups to leave the school grounds either to go home to eat or to buy lunch from various outlets, which can result in the consumption of fast food, energy drinks or other unhealthy or energy-dense foods.^(41)^

This work has several additional strengths. To our knowledge, it is the first systems map of the UK secondary school food system, co-developed with stakeholders to reflect their lived experiences. The inclusion of secondary school students in the process is a particular strength, as it ensured that the map reflected the experiences of those at the heart of the problem. There were some stakeholder voices which were not captured either in the online survey or in the group model building workshop. Whilst we had teachers participate, none of these teachers identified as senior leadership within the schools, meaning we didn’t have the voices of headteachers. As the decisions for a school will so often be led by headteachers, we consider this a limitation of the study. It could also be seen as a limitation that our map was developed with qualitative methods and thus does not demonstrate relative strength of factors, relationships and feedback loops. Future research could collect quantitative data from stakeholders to provide insight into the relative strengths of relationships. Future work could also explore feedback loops in more depth, identifying those which shape and sustain food choice patterns in secondary schools, and also include all regions and stakeholder groups to ensure a fully comprehensive systems map of the UK secondary school food system.

### Conclusion

The study used participatory methods with school stakeholders to co-produce a systems map of the UK secondary school food system. The map highlights the complexity of the system and the dynamic relationships which influence food choice at school. Visualising the system in this way helps identify impactful action and interventions and can aid stakeholders in designing and evaluating whole-school, systems-informed, multi-component interventions and programmes targeting food choice in secondary schools.

## Supporting information

O’Kane et al. supplementary material 1 O’Kane et al. supplementary material

O’Kane et al. supplementary material 2 O’Kane et al. supplementary material

O’Kane et al. supplementary material 3 O’Kane et al. supplementary material

O’Kane et al. supplementary material 4 O’Kane et al. supplementary material

## Supplementary material

For supplementary material accompanying this paper visit https://doi.org/10.1017/S136898002510147X
