# Supplementary material for: Factors contributing to food choice in the UK secondary school food setting: a systems map perspective
Source: Public Health Nutr. 2025 Dec 3;28(1):e208. doi: 10.1017/S136898002510147X (PMC12809607; doi:10.1017/S136898002510147X)
Supplement: O’Kane et al. supplementary material 1 — O’Kane et al. supplementary material [file S136898002510147Xsup001.docx]

**Online survey questions**

Q1. What is your role within the secondary school food system?

> Drop down list: Teacher, headteacher, secondary school student, catering staff, local authority, parent/carer, school governor, policy maker, other (please specify)

> Other:

Q2. Where in the United Kingdom are you located?

> Drop down list: Northern Ireland, Scotland, Wales, England, other (please specify)

> Other:

Q3. We are interested in identifying different factors which impact upon the secondary school food system and the resulting food choice for secondary school students during the school day.

Examples might include: ‘parent’s opinion’, ‘closeness to fast food restaurants’, ‘food offered in the canteen’, ‘what students like to eat’. You can also include these examples if you think they are important factors.

Please identify factors you believe drive food choice within the secondary school food system
NB: Please suggest a **minimum of 5** factors, and up to a **maximum of 15**

Q4. [Optional] If you have anything else you would like to let us know about the above, please do so below

[Open text box]
