# Supplementary material for: Factors contributing to food choice in the UK secondary school food setting: a systems map perspective
Source: Public Health Nutr. 2025 Dec 3;28(1):e208. doi: 10.1017/S136898002510147X (PMC12809607; doi:10.1017/S136898002510147X)
Supplement: O’Kane et al. supplementary material 2 — O’Kane et al. supplementary material [file S136898002510147Xsup002.docx]

**Results from the online survey**

Table 1. Factors identified by survey respondents

| Price |
| --- |
| Peer acceptability |
| Familiarity |
| Quick to eat |
| Filling |
| Perceptions of students preference for fast food |
| Cost to school / catering company |
| Serving students quicky in limited time period |
| Food that is quick to eat |
| Price point to students |
| Price for each meal provided |
| Choice of dishes |
| Vegetarian choices |
| Air miles |
| I would like seasonal foods |
| Local providers |
| Quality of product |
| Reduce highly processed foods |
| More fruit and vegetables |
| Quantity i am not a big eater and always ask for smaller portions |
| Less sugar in products |
| We have a no salt policy at school |
| Portion sizes |
| Cultural appropriate |
| Cost |
| Where its prepared |
| Eating environment |
| Time allowed |
| Catering for different diets |
| Range offered |
| Does it encourage young people to try new things |
| Peer pressure |
| Appearance |
| Commercial options nearby |
| Price |
| Seating available for meals |
| Pupil voice |
| Time available for eating a meal |
| Ingredient availability |
| School food standards compliance |
| Lunchtime clubs and activities |
| Queuing / speed of being served different items |
| Appearance/marketing of the food |
| What peers are choosing e.g. Packed lunch vs school meal, hot vs 'grab and go' |
| Whether school has an open policy at lunchtime (for pupils to buy food elsewhere) |
| Equity of access for pupils eligible for free school meals (can they access everything that their peers can/are there opportunities for stigma) |
| Quality of the food |
| Availability of popular food items |
| Placement/nudging of particular items |
| Whether catering contract is negociated by school or consortium/local authority |
| Individual school (and local authority) engagement with healthy and environmentally sustainable food / menus |
| Level of pupil engagement in menu planning/consultation and feedback (e.g. What they like/don't like, suggestions for improvement) |
| Time allowed for consumption of food |
| Spaces allowed for consumption of food |
| Dietary requirements |
| Cost |
| Nutritional values |
| Sustainability |
| Local sourcing |
| Time allowed for socialising with friends during lunch bteak |
| Teachers / staff eating with students |
| Diversity of cuisine ethnicity |
| Limiting sugary foods |
| No artificial sweeteners permitted |
| Water readily available |
| Reuseable cups for water readily available |
| Food prepared on premises |
| Timetabling and break structure |
| Lack of time |
| Pupils being allowed off site - access to shops and takeaways |
| Lack of whole school food policy and positive food culture |
| No monitoring of school food standards |
| Financial - schools want to offer items high in fat, sugar and salt as popular and increase income |
| Catering and school staff lack of knowledge |
| Schools following other schools - downward spiral of poor quality/unhealthy food |
| Pupils lack of knowledge |
| School meals funding too low - caterers restricted by low budgets |
| Suppliers promoting "school compliant" unhealthy items |
| Supply issues |
| Food education not embedded into curriculum |
| Lack of dining facilities |
| Peer pressure/influence |
| Cost of food |
| Catering practices within school |
| School leadership |
| Pupilsâ€™ preferences |
| Ease of preparation |
| Cost, ensuring value for money |
| Variety of the food offerings available |
| Time - to purchase and consume foods within school settings |
| The speed and efficiency of food service |
| The environment to purchase and consume food items |
| The demographics of the student populace int terms of healthy eating importance |
| The influence of advertising and social media on food habits |
| Parent/ carer influences/ opinions on healthy eating |
| The ethos of an individual school/ federation |
| Food on offer |
| Ease of access to food on offer (queues) |
| Ease of payment |
| Principal's views |
| School's food policy |
| Enforcement of school policy |
| Visibility of school policy |
| Pupil's nutritional knowledge |
| Parent's attitude towards food and healthy choices |
| Peer behaviour |
| Nutritional standards that are food based and can be implemented |
| Supports available to ensure standards are translated into a food offering that appeals to young people |
| Visibility of importance of school food |
| Government commitment to school food through food in schools forum |
| Type of foods available from procurement framework |
| Stay on site policy |
| Stigma associated with free school meals |
| That school meals are not universal |
| Tension between those who do/do not avail of school food |
| Disjoint between standards and other aspects of food in school |
| Cinderella status of food and cooking skillsre of |
| Tension between education (curriculum) and health (food environment) |
| Quantity and quality of evidence on school food |
| Sharing of findings/experiences including things that don not work well |
| Data on school food metrics for each country/area |
| Prefer hand held/fast food i.e panini, wraps, pizza |
| Value/easy on the purse |
| School too close to sweet shop, before and after school |
| What their friends are eating |
| What foods they receive at home |
| Not liking the look of something without trying it first |
| The weather |
| Other children selling drinks/sweets, purchased from outside of school |
| Being restricted by nutrional standards on what meats, diary they can eat throughout the week |
| Are not fussed on vegetables |
| Food offered in school canteen, vending etc |
| Pupil likes and dislikes |
| Food brought to school in packed lunch, snacks etc |
| Access to shops on way to school |
| Money available to pupils |
| Family income |
| Food knowledge |
| Interest in health |
| Involvement in sport |
| Stress levels - comfort eating, sugar rush |
| Unattractive presentation of food |
| Balancing food intake and play time at break and lunch |
| Food environment eg noise levels in canteen may discourage some pupils |
| Food prices - f and v more expensive than â€˜junk foodâ€™ makes healthy eating difficult |
| Profit over health prioritised be canteen and vendors |
| The child in post primary education should be able to decide for themselves if the wish to eat a healthy dish or something less healthy |
| I feel nutritional standards have taken the joy out of our service |
| We have lots of garages that have deli counters in our area. If the child doesn't get what it wants in school it goes to the garage |
| Food offered in our canteens can be limited due to the standards |
| Compiling menus are difficult when trying to meet guidlines |
| Personal prefernce of pupil |
| What is offered in dining hall |
| Proximity to local centra convenience store |
| Cost |
| Food provided by parents |
| Food in schools policy |
| Cost |
| Likes and dislikes of pupils |
| Free school meals |
| Whats on offer |
| Nuttritional standards |
| Cost |
| Popularity |
| Trends in eating eg street food |
| Availability of supplies |
| Season |
| Ambience |
| Q jumping |
| Popular foods |
| Able to eat fast |
| Students taste |
| Easy to bulk prep |
| Parents/teacher choice |
| Choice |
| What other pupils are eating |
| Time available |
| Price |
| Hunger |
| The ordering system |
| Time |
| Students appetite |
| Cost |
| Healthy food |
| Whole foods |
| No processed foods |
| Proper nutrition |
| Less sugar |
| More quality natural fats |
| What pupils like to eat |
| Taste |
| Portion size |
| Speed of access (queues and short lunchtime) |
| Cost |
| Peer pressure |
| Time |
| Proximity |
| Parent influence |
| Health |
| Medical conditions |
| Education |
| Staff |
| Peers |
| Location of other food outlets |
| Cost |
| Not getting selection that they wish for |
| Choices available |
| Other outside options available to them |
| Cost |
| Vending machine illegal |
| Principal non participation |
| Presentation |
| Theme days |
| Fast food options |
| Healthy options |
| Prices |
| Popularity of food available |
| Types of foods offered |
| Personal preferences |
| Parental influence |
| Time of year |
| Healthy eating habits |
| Cost of food available |
| Peer pressure |
| Chips |
| Snack items burgers etc |
| Price value for money |
| Salad bar |
| Seating arrangements |
| Food choices |
| Catering staff friendly |
| Homemade food |
| Food offered in canteen |
| Quality of food |
| Variety of choice |
| Use of snack bar |
| Cost of food |
| Proximity to local fast food |
| Proximity to local deli |
| Proximity to local supermarket |
| Fast to consume |
| Peer opinion on your choice |
| Eating is cheating |
| Want more |
| Tastes great |

Table 2. Survey respondents

| Parent/carer | 15 |
| --- | --- |
| Catering staff | 4 |
| Local authority | 3 |
| School food researcher | 2 |
| Teacher | 1 |
| School support staff | 1 |
