# Supplementary material for: Factors contributing to food choice in the UK secondary school food setting: a systems map perspective
Source: Public Health Nutr. 2025 Dec 3;28(1):e208. doi: 10.1017/S136898002510147X (PMC12809607; doi:10.1017/S136898002510147X)

**Plan for group model building workshop**

**Problem of interest:**

What are factors, the inter-relationships between these factors, that shape food choice in secondary school settings in Northern Ireland?

**Objectives:**

1. Build shared understanding of the complex system influencing food choice in secondary school settings in Northern Ireland.
2. Identify and explore priorities, opportunities, and actions to improve food choice in secondary school settings in Northern Ireland, reflecting on what is of value to stakeholders and what is needed to implement the actions.

**Outputs:**

1. Causal loop diagram of the complex system influencing food choice in secondary school settings in Northern Ireland. (see an example at the end of this document)
2. Preliminary list of set of priorities, opportunities, and actions to improve food choice in secondary school settings in Northern Ireland, informed by the causal loop diagram.

**Core modelling team (responsible for designing the workshop):**

| **Role** | **Name** |
| --- | --- |
| General oversight | Leandro Garcia, Ruth Hunter |
| Scientific objectives | Niamh O’Kane, Jayne Woodside |
| GMB process | Leandro Garcia, Ruth Hunter |
| Modeller | Leandro Garcia |
| Familiarity with stakeholders | Niamh O’Kane, Jayne Woodside |
| Logistics | Niamh O’Kane, Leandro Garcia |

**Facilitation team (responsible for delivering the workshop):**

| **Role** | **Name** |
| --- | --- |
| Meeting convener/closer | Jayne Woodside |
| Facilitators | Ruth Hunter, Leandro Garcia |
| Modeller | Leandro Garcia |
| Stage manager and timekeeper | Niamh O’Kane |
| Note taker | Niamh O’Kane, Jayne Woodside |

**Attendees:**

- Local stakeholders representing multiple sectors, such as governmental agencies, school community (i.e., board of governors, teachers, parents, students), advocacy groups, and researchers.

**Logistics and preparation for the workshop:**

- Virtual meeting platform: Zoom
- Modelling platform: STICKE
- Transition and action ideas platform: Mural
- Prepare participants for the workshop (e.g., materials and link for the day).

**Agenda (2h30min)**

| **Clock time** | **Duration** | **Activity** | **Leads** | **Description** |
| --- | --- | --- | --- | --- |
|  | 10 min | Welcome and introductions | Jayne Woodside | - Introduction of facilitation team - Summary of workshop goals - Agenda for the day - Expectations and commitments - Note taking – we will have note takers. If someone shares something and do not want it recorded, talk to the notetaker after the session - Informed consent |
|  | 5 min | Problem articulation | Jayne Woodside | - Define problem to be addressed in the workshop |
|  | 10 min | Introduction to systems thinking and CLD | Leandro Garcia | - Introduction to systems thinking and causal loop diagrams |
|  | 45 min | Connection circle and CLD structure | Leandro Garcia  Ruth Hunter | - Connect factors raised in the survey - Generate the CLD structure |
|  | 10 min | Break (modeller will be working to finish up the causal loop diagram) | | |
|  | 20 min | Model review | Leandro Garcia  Ruth Hunter | - Overview of the causal loop diagram - Verify the causal loop diagram together with participants in case there is something that needs to be added, removed, or changed |
|  | 40 min | Action ideas | Ruth Hunter  Leandro Garcia | - Identification and prioritization of actions to change the system |
|  | 10 min | Next steps and closing | Jayne Woodside | - Explain what is next - Thank participants |

See detailed plans for each activity next.

**Welcome and introductions**

- Platforms: Zoom
- Primary nature of group task: not applicable
- Inputs: none
- Outputs:
  - Facilitation team introduced
  - Workshop participants introduced
  - Objectives, expectations, and agenda of the day introduced
- Roles:
  - Meeting convener/closer: Jayne Woodside
  - Stage manager and timekeeper: Niamh O’Kane
- Steps:
  - Meeting convener/closer will welcome participants and introduce:
    - The project
    - The facilitation team
    - Workshop objectives
    - Agenda of the day
  - Participants introduce themselves.
  - Explain that Niamh O’Kane will be taking notes. Notes will be anonymised, but if someone shares something and do not want it recorded, talk to the facilitation team after the session.
  - Remember of the informed consent.
  - Ask if there are any questions or comments.

**Problem articulation**

- Platforms: Zoom
- Primary nature of group task: not applicable
- Inputs: none
- Outputs: shared understanding of the problem of interest
- Roles:
  - Presenter: Jayne Woodside
  - Stage manager and timekeeper: Niamh O’Kane
- Steps:
  - Presenter to briefly present key aspects of food choice in secondary school settings in Northern Ireland.
  - Presenter to introduce the problem of interest, making clear long-term goals and visionary future but to also consider short- and middle-term goals and actions.
  - Ask if there are any questions or comments.

**Introduction to systems thinking and causal loop diagram**

- Platforms: Zoom
- Primary nature of group task: not applicable
- Inputs: none
- Outputs:
  - Understanding of the underlying perspectives that will be used in the workshop
- Roles:
  - Presenter: Leandro Garcia
  - Stage manager and timekeeper: Niamh O’Kane
- Steps:
  - Presenter will introduce systems thinking and key elements necessary to read a causal loop diagram (nodes, links, link polarity, reinforcing and balancing feedback lops, time delays).
  - Ask if there are any questions or comments.

**Connection circle and CLD structure**

- Platforms: Zoom (meeting) + STICKE (modelling)
- Primary nature of group task: divergent
- Inputs: factors elicited in the survey
- Outputs:
  - Causal loop diagram
- Roles:
  - Modeller: Leandro Garcia
  - Facilitator: Ruth Hunter
  - Notetaker: Niamh O’Kane and Jayne Woodside
  - Stage manager and timekeeper: Niamh O’Kane
- Steps:
  - Modeller presents the circle with factors elicited in the survey. The task is to draw between pairs of variables connections that are important to shape the system affecting food choice in secondary school settings in Northern Ireland.
  - (time for instructions: 5 minutes) Participants individually have 5 minutes to connect pairs of variables:
    - Ask participants to pick two variables that are connected and draw a line with an arrow pointing in the direction of influence. Then repeat with as many pairs of variables as they wish for 5 minutes. Say that the arrow shows causality and it can indicate both a positive or a negative situation. Provide an example to the participants.
    - If a pair of variables has connections in both ways, draw one arrow for each direction.
    - Arrows can go from “food choices” to other factors too.
    - Concentrate in connections that indicate causality and that are important drivers or consequences of food choice in secondary school settings.
    - They can do it in a Word or PowerPoint file or a piece of paper.
    - Tell participants that they will share the connections they draw with the wider group to form a shared causal loop diagram, so they should think which of their connections are more and less critical to include in the final diagram.
  - For the next 30 minutes, participants will take turn sharing 1-2 connections that are not already in the diagram. Clarifications can be asked and disagreements can be expressed and discussed by the participants, but the facilitator will guide the discussion to ensure orderly participation in the activity. The modeller will add the connections.
  - In the last 5 minutes, the modeller will transform the connection circle into a preliminary causal loop diagram and let the participants to glance at it.
  - The notetakers document insights shared that were not captured in the diagram.
  - Over the 10-min break that will follow this activity, the modeller will adjust the causal loop diagram for best visualization.

**Model review**

- Platforms: Zoom (meeting) + STICKE (modelling)
- Primary nature of group task: convergent
- Inputs: preliminary causal loop diagram from the “Connection circle and CLD structure” activity
- Outputs:
  - Revised causal loop diagram
  - List of main feedback loops and dynamics identified
  - Shared understanding of the system and insights that have emerged
- Roles:
  - Modeller: Leandro Garcia
  - Facilitator: Ruth Hunter
  - Notetaker: Niamh O’Kane and Jayne Woodside
  - Stage manager and timekeeper: Niamh O’Kane
- Steps:
  - Modeller describes main aspects of the causal loop diagram.
  - Facilitator and modeller then initiate questioning regarding what did not get captured or is missing from the diagram. Modeller changes the diagram accordingly, sharing their screen to allow participants to see the changes as they are implemented.
  - The facilitator confirms the adequacy of the diagram as a representation of the group thinking, points out important changes in the model’s structure as they happen, and highlight insights that emerge.
  - The notetakers document the insights shared.

**Next steps and closing**

- Platforms: Zoom
- Primary nature of group task: not applicable
- Inputs: none
- Outputs: participants informed of what the next steps with the model and the project are
- Roles:
  - Meeting convener/closer: Jayne Woodside
  - Stage manager and timekeeper: Niamh O’Kane
- Steps:
  - Meeting convener/closer thanks participants for their contributions during the session and explains what it is going to happen next with the diagram and the project:
    - Clean-up and review: tell participants that the diagram will be cleaned up by comparing what is currently included in the model to the notes, and everything that was said will be incorporated into the model. The diagram and the output of the action ideas session will be sent to all participants once the clean-up and review is finished.
    - Summarize next steps.
  - Ask if there are any final questions or comments, and state how participants can reach out to us.
  - Close the meeting

**Example of causal loop diagram**


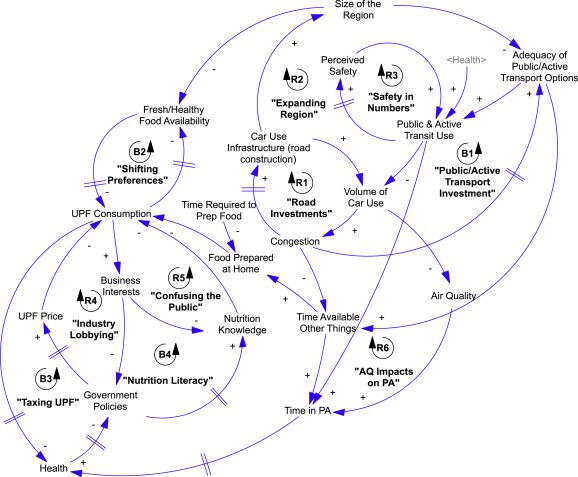


Causal loop diagram of the system that influences food behaviours and transport in 3 Latin American cities. From “Using community-based system dynamics modelling to understand the complex systems that influence health in cities: The SALURBAL study” (<https://doi.org/10.1016/j.healthplace.2019.102215> - open access). The + and - signals beside the arrows indicate that an increase in factor A is expected to lead to an increase or decrease, respectively, in factor B. The “||” symbol on an arrow represents a time delay in the relationship between two factors. “R” identifies reinforcing feedback loops. “B” identifies balancing feedback loops. Table 5 below list and describe the key feedback loops (something we will do too). AQ = air quality. PA = physical activity. UPF = ultra-processed food.


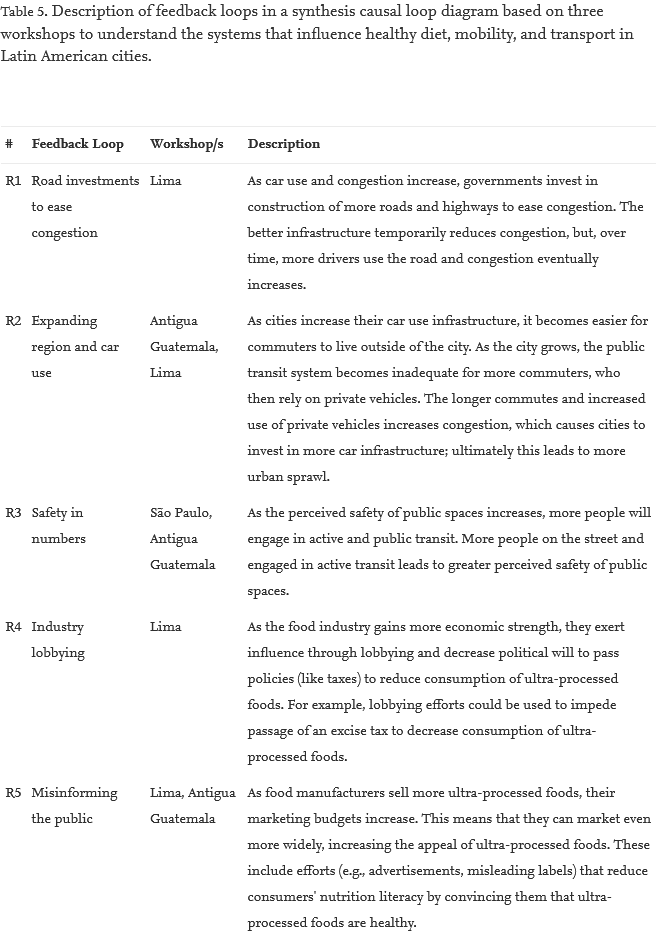


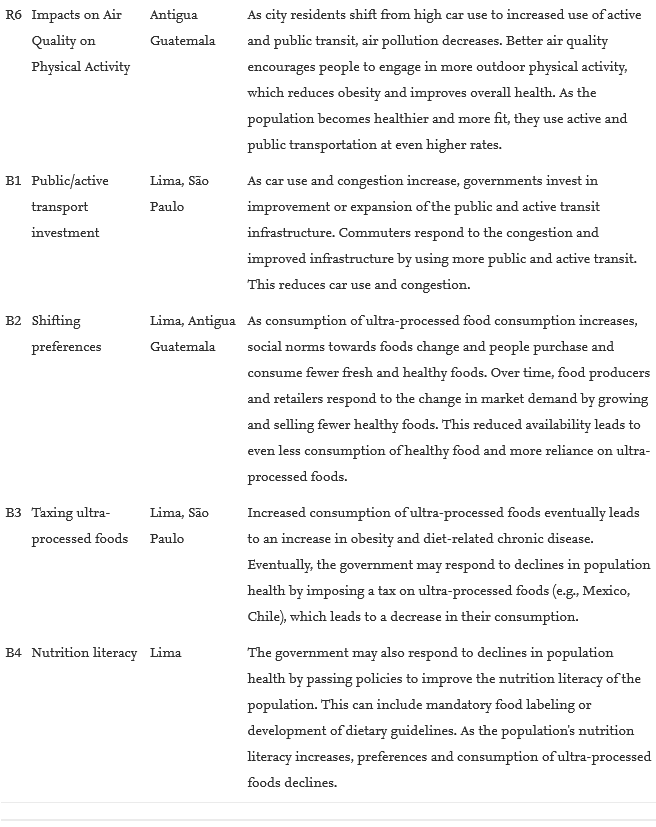

Supplement: O’Kane et al. supplementary material 3 — O’Kane et al. supplementary material [file S136898002510147Xsup003.docx]
