# Supplementary material for: Factors contributing to food choice in the UK secondary school food setting: a systems map perspective
Source: Public Health Nutr. 2025 Dec 3;28(1):e208. doi: 10.1017/S136898002510147X (PMC12809607; doi:10.1017/S136898002510147X)
Supplement: O’Kane et al. supplementary material 4 — O’Kane et al. supplementary material [file S136898002510147Xsup004.docx]

# Node names and glossary

## Healthier food choice at school

“Outcome variable”

## Presentation and taste of food

- Presentation/appearance of food
- Food looking appealing
- Taste of food
- Food architecture, placement and labelling (incidental learning)
- Nudging and marketing
- Portion size

## Procurement and availability of supplies

- Type of foods available from procurement framework
- Availability of supplies/supply issues
- Suppliers promoting "school compliant" unhealthy items

## Provision and selection of foods that is on offer

- Food offered in school canteen
- Theme days
- Vegetarian options
- Diversity of cuisine, culturally appropriate
- How “healthy” this food is
- Food offered in our canteens can be limited due to the standards
- Compiling menus are difficult when trying to meet guidelines
- Food must be easy to bulk prep
- Ease of preparation
- Popularity of food able to eat fast, e.g. grab-n-go, hand held/fast food i.e Panini, Wraps, Pizza
- Catering practices within school

## Concerns about sustainability

- Sustainability
- Local suppliers
- Air miles

## Time to eat

- Time allowed to eat (and socialise) during breaks
- Balancing food intake and play time at break and lunch
- Time allowed for socialising with friends during lunch break
- Lunchtime clubs and activities taking place during time allocated to eat

## Eating environment

- Speed of queues/access
- Seating arrangements
- Canteen noise
- Lack of dining facilities
- Spaces allowed for consumption of food

## Food embeddedness in the curriculum

- Visibility of importance of school food
- Tension between education (curriculum) and health (food environment)
- Food education not embedded into curriculum

## Pupil’s food knowledge

- Pupil's nutritional knowledge
- Pupil's knowledge about food
- Pupil’s interest in health/involvement in sport

## Policy implementation and nutritional standard compliance

- Visibility of food policy
- Enforcement of food policy
- Food in schools policy
- Lack of Whole School Food Policy
- Food meeting nutritional standards
- School food standards compliance
- Food offered in our canteens can be limited due to the standards

## Food environment at home

- Parent/carer behaviour and attitudes
- Parent's attitude towards food and healthy choices
- Foods eaten and provided at home
- Parents and teachers choosing foods
- Food brought to school in packed lunch, snacks etc.

## Positive school food culture

- Positive food culture
- The ethos of an individual school/federation

## School leadership commitment to food

- Government commitment to school food through food in schools forum
- Principal non participation
- Whether catering contract is negociated by school or consortium/local authority
- School leadership influence

## Appeal of and proximity to local shops and food outlets

- School's proximity to delis, shops, fast food
- Stay-on-site vs open policy at lunchtime

## Efficiency of selection/purchasing

- The ordering system in place for selection/purchasing
- Ease of payment
- Speed of access
- Nudging and placement of items

## Catering/school staff knowledge of food and food skills

- Catering and school staff knowledge (or lack thereof)

## Pupil’s appetite

- Child temperament
- Mental health
- Hunger and appetite
- Stress levels - comfort eating, sugar rush

## Pupil’s usual healthy eating habits and behaviours

- Pupil’s interest in health/involvement in sport
- Restrictive food behaviours (e.g., "eating is cheating")
- Dislike/avoid vegetables
- Degree of healthiness of foods consumed
- Pupil's likes and dislikes
- Medical conditions
- Familiarity with foods
- Dietary requirements

## Healthy behaviour/influence of friends, peers, social circle

- Peer behaviour and influence of social circle
- What their friends and others are eating
- Popularity
- Other children selling drinks/sweets, purchased from outside of school
- Tension between those who do/do not avail of school food
- The influence of advertising and social media on food habits
- Trends in eating (e.g., street food)

## School meals funding

- Schools may offer items high in fat, sugar, and salt as popular and increase income
- Profit over health prioritised be canteen and vendors
- School meals funding too low

## Cost of food on offer

- Price of food
- Price point for students
- Food that is value for money
- Food prices - f and v more expensive than 'junk food' makes healthy eating difficult

## Family income

- Money available to pupils
- Eligibility for free school meals
- Food poverty

## Uptake and access of free school meals

- Stigma associated with free school meals
- Equity of access for pupils eligible for Free School Meals (can they access everything that their peers can/are there opportunities for stigma)
